# Effects of Baduanjin imagery and exercise on cognitive function in the elderly: A functional near-infrared spectroscopy study

**DOI:** 10.3389/fpubh.2022.968642

**Published:** 2022-09-29

**Authors:** Lianqiang Yao, Guoxiao Sun, Jun Wang, Yujuan Hai

**Affiliations:** School Physical Education, Shandong University, Jinan, China

**Keywords:** Baduanjin, motor imagery, the elderly, cognitive function, fNIRS

## Abstract

**Objective:**

Cognitive function is essential in ensuring the quality of life of the elderly. This study aimed to investigate the effects of Baduanjin imagery and Baduanjin movement (a traditional Chinese health exercise, TCHE) on cognitive function in the elderly using functional near-infrared spectroscopy (fNIRS).

**Methods:**

72 participants with a mean age of 66.92 years (SD = 4.77) were recruited for this study. The participants were randomly assigned to three groups: the Baduanjin imagery, the Baduanjin exercise, and the Control. Stroop task was used to record the accuracy and reaction times, and a near-infrared spectral brain imaging system was used to monitor the brain's oxy-hemoglobin concentration responses.

**Results:**

(1) For the reaction times of Stroop incongruent tasks, the main effect of the test phase (*F* = 114.076, *p* < 0.001) and the interaction effect between test phase and group (*F* = 10.533, *p* < 0.001) were all significant. The simple effect analysis further demonstrated that the reaction times of the Baduanjin imagery group and Baduanjin exercise group in the post-test was faster than that in the pre-test (*ps* < 0.001); (2) Analysis of fNIRS data showed the significant interaction effect (*F* = 2.554, *p* = 0.013) between the test phase and group in the left dorsolateral prefrontal cortex. Further analysis showed that, during the post-test incongruent tasks, the oxy-Hb variations were significantly higher in participants of the Baduanjin imagery group (*p* = 0.005) and Baduanjin exercise group (*p* = 0.002) than in the control group; For the right inferior frontal gyrus, the interaction between the test phase and group was significant (*F* = 2.060, *p* = 0.044). Further analysis showed that, during the post-test incongruent tasks, the oxy-Hb variations were significantly higher in participants of the Baduanjin imagery group than in the control group (*p* = 0.001).

**Conclusion:**

Baduanjin imagery and exercise positively affect cognitive performance; Baduanjin imagery and exercise activated the left dorsolateral prefrontal cortex; Baduanjin imagery activated the right inferior frontal gyrus, while Baduanjin exercise could not.

## Introduction

According to China's National Bureau of Statistics, by 2020, there are already 260 million people aged 60 years and older in China, accounting for 18.7% of the total population (1). Improving the quality of life and well-being of the elderly has attracted more and more attention in academia due to the high level of population aging. The decline in mental status brought about by aging is one of the most critical factors affecting the quality of life of the elderly. The ability of the elderly to live independently and social well-being is closely related to brain function (2, 3). With the decline of brain function, especially in the frontal cortex area, older adults show a downward trend in daily cognitive abilities (4, 5), including working memory, attentional control, imagination, and decision-making. Therefore, the search for simple and feasible methods to improve brain function in the elderly has become one of the current research hotspots. Due to cognitive and neurological plasticity (6), we can activate the cerebral cortex and improve the functional connectivity of brain areas through exercise training, cognitive training, and psychological interventions, thus slowing down the rate of brain decline and improving the cognitive function of the elderly (7, 8).

Numerous studies show that physical exercise positively affects cognitive and motor function in older adults (9–12). Baduanjin is a traditional Chinese health exercise (TCHE) (8), different from the general resistance exercise, endurance exercise, and strength exercises, emphasizing the coordination of movements and breathing and concentration attention (13, 14). It is suitable to improve the physical and mental health of the elderly. The Baduanjin has the advantages of being soothing, simple, and high safety and is popular among the elderly at home and abroad (13). Research has confirmed that Baduanjin is suitable for muscle strength improvement (15) and sleep improvement (7, 16), as well as for improving cognitive function (8, 17).

In addition to traditional exercise interventions to improve physical and mental health, another widely researched and applied approach is motor imagery training, which has the characteristics of high safety and operability (18–20). As a form of psychological intervention, motor imagery does not require real action but only simulates movement within the mind and reflects the brain's perceived movement (21), which can be applied in the case of physical inconvenience or limited movement space. Previous studies have shown that motor imagery has neurophysiological similarities to actual movement. For example, Jacobson (22) suggests that people have similar peripheral physiological effects during motor imagery as actual movements, such as action-potentials and heart rate. Motor simulation theory proposed by Jeannerod (21) argues that motor imagery is functionally equivalent to actual movement in terms of the planning and engagement of neural circuitry (23). The neural similarities have prompted researchers to keep exploring more possible applications of motor imagery, such as improving motor function [e.g., walking speed (19), muscle strength (15)] or improving cognitive function (18, 20) (e.g., attention, working memory). As one of the motor imagery, the Baduanjin imagery is supposed to have the same facilitating effect on improving the brain's cognitive decline.

The prefrontal lobe (PFC) is an important brain region for executive functions, with the dorsolateral prefrontal (DLPFC) and the inferior frontal gyrus (IFG) being the main activation areas (24, 25). They have an essential role in ensuring attention allocation and enhancing cognitive control, which are the important brain regions in the cerebral cortex responsible for engaging and maintaining the attentional demands of individuals (25–27). Studies showed that physical exercise increases blood oxygen concentrations in the left DLPFC (17, 26, 28), and Malouin (29) also found that motor imagery increased participants' left DLPFC blood oxygen concentrations as the actual movement. In addition, many neuroimaging researches demonstrated that the exercise (30–32) and motor imagery (33–35) could also activate the IFG. Therefore, to further elucidate the neural mechanisms by which motor imagery and exercise improve cognitive function, it is necessary to explore the activation effect of motor imagery and exercise on the left DLPFC and the right IFG.

Functional near-infrared spectroscopy (fNIRS) is widely adopted in cognitive neuroscience to measure the cortical oxy-hemoglobin concentration in real-time, reflecting the individual brain activation status (36). It is an optical neuroimaging technique based on the neurovascular coupling and optical spectroscopy theory (37, 38), which focuses on monitoring changes in blood oxygen in the brain by spectroscopic determination of oxy-hemoglobin and deoxy-hemoglobin and quantitatively describes light transport in tissue to study neural mechanisms involved in cognitive activity (39). Previous studies have shown that fNIRS can be used to assess the brain hemodynamic characteristics of able-bodied and impaired cohorts populations (38), such as those with mild cognitive impairment, Alzheimer's disease, depressive disorders, and stroke, facilitating early detection of cognitive impairment and monitoring of rehabilitation effects. Because of its portability, safety, low cost, and high temporal resolution (39), fNIRS is well suited to studying the effects of exercise on cognitive performance and brain oxygenation/hemodynamics. These advantages make fNIRS has the potential for widespread implementation in exercise–cognition science research (37) and for investigating the brain neural mechanisms by which exercise affects the cognitive function in older adults (40). In addition, motor imagery-related studies have mainly used cognitive behavioral tasks to evaluate the effect on improving cognitive function (41, 42). It is necessary to further investigate its neural mechanisms using neuroimaging techniques. Therefore, we aimed to measure blood oxygen in the inhibition control tasks of the elderly to evaluate the effect of Baduanjin imagery on neuronal activity in the left DLPFC and the right IFG by fNIRS.

The current study monitored changes in the PFC blood oxygenation in the elderly before, during, and after the intervention using fNIRS. We aimed to explore whether Baduanjin imagery has the same effect as actual movement and examine the mechanism of cerebral blood oxygenation in improving cognitive function by Baduanjin imagery to provide a practical basis for enhancing cognitive function in the elderly.

We set up the Baduanjin imagery group, the Baduanjin exercise group, and the Control group and proposed the following hypotheses: (1) Baduanjin imagery improves cognitive function in the elderly, as reflected in the Stroop task reaction times; (2) Baduanjin exercise improves cognitive function in the elderly, as reflected in the Stroop task reaction times; (3) Baduanjin imagery practice enhances oxy-hemoglobin concentration in the left DLPFC and the right IFG during the inhibition task; (4) Baduanjin exercise enhances oxy-hemoglobin concentration in the left DLPFC and right IFG during the inhibitory control task.

## Materials and methods

### Participants

According to G-power 3.1 (43), to estimate the experimental sample size, concerning the medium effect size *f* = 0.25 and power 1-β = 0.95, we calculated the minimum sample size to be 66 people and planned to recruit 70 people considering sample attrition. Furthermore, we recruited 76 participants from the local community in Jinan, China, of whom one quit without completing the experiment, one slept during the intervention, and two had a low ACC rate (9.26 and 13.89%). Finally, data from 72 participants (60–75 years old with a mean age of 66.92 years, SD = 4.77) were included in the data analysis. The participants were randomly assigned to the Baduanjin imagery group, the Baduanjin exercise group, or the Control group. The eligibility criteria were age 60 and above, having Baduanjin exercise experience ≥3 years, and MMSE score ≥24. The exclusion criteria were the presence of neurological disease, vision problems, or previous similar experimental experiences. This study was previously approved by the Institutional Ethics Committee. All participants signed informed consent before the experiment.

### Materials

#### Mini-mental status examination (MMSE)

The MMSE is prepared by Folstein (44) and revised by Li (45). It includes questions on time and place orientation, attention and calculation, recall, language, and visual construction. The scale ranges from 0 to 30. The study was excluded the participants wihe scores ≤ 23 indicating cognitive impairment. The Cronbach's alpha of the Chinese version of MMSE is 0.97 (45).

#### Physical activity rating scale-3 (PARS-3)

The PARS-3 revised by Liang and Liu (46) includes activity intensity, time, and frequency by the 5-point Likert scale. Physical activity score = intensity score × frequency score × (time score – 1). The Cronbach's alpha is 0.796 (47).

#### Movement imagery questionnaire-revised (MIQ-R)

The ability to kinesthetic and visual imagery was assessed using the MIQ-R revised by Zhang and Mao (48). The questionnaire consisting of 2 subscales, each with four questions, asked subjects to complete actions by motor imagery and evaluate the difficulty of completing these mental tasks on a 7-point Likert scale. The Cronbach's alpha for the kinesthetic and visual imagery subscales were 0.75 and 0.83 (48), respectively.

#### Cognitive task

**Figure 1:**
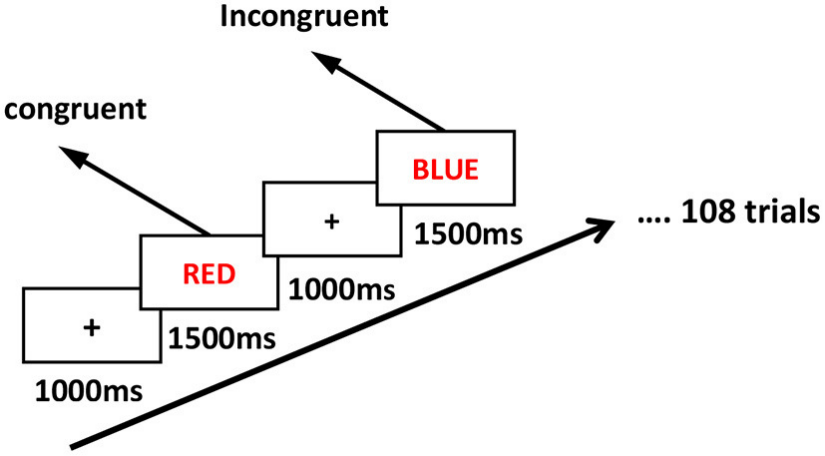
The Stroop task paradigm. The entire Stroop task consists of a practice task consisting of 15 trials and a formal task composed of 108 trials (36 incongruent, 72 congruent).

We used the Stroop test as the cognitive test (26, 49), which was programmed with E-Prime 3.0 (Psychology Software Tools). The test required the participants to discriminate whether the color of the Chinese word matched the meaning of the words. Judgments were made with numeric keyboard responses of “1” and “2” with the right index and middle fingers, respectively, for the congruent condition (such as the word “RED” shown in red color) and the incongruent condition (such as the word “BLUE” shown in red color). The test includes a practice task consisting of 15 trials and a formal task composed of 108 trials (36 incongruent, 72 congruent), consisting of 1500 ms simulations followed by an inter-stimulus interval of 1000 ms (Figure 1). The behavioral task results include reaction times (RTs) and accuracy (ACC).

### Procedures

**Figure 2:**
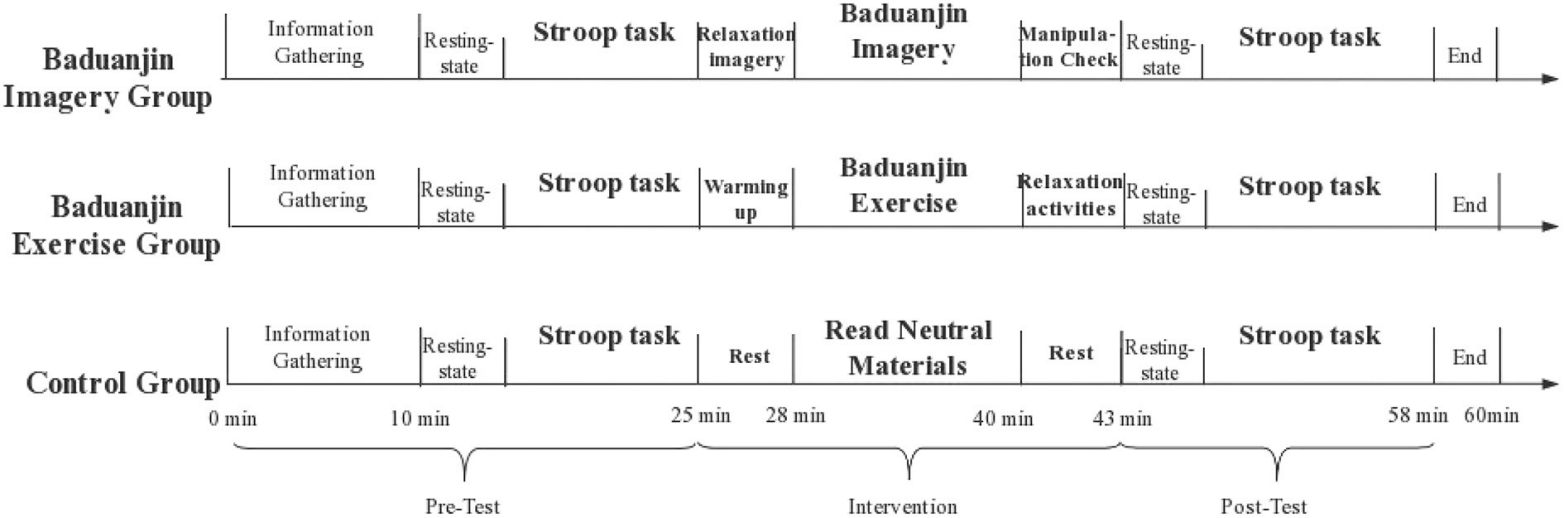
Chart of the experimental protocol.

The experimental protocols included a pre-test, an intervention, and a post-test (Figure 2). In the pre-test, participants were informed of the content and procedure of the experiment upon arrival at the laboratory, filled out an informed consent form, the demographic questionnaire, the PARS-3 assessing recent activity, the MMSE assessing cognitive status, and the MIQ-R assessing individual imagery ability, and then completed the tests.

In the intervention, three groups of participants performed the Baduanjin imagery training, the Baduanjin exercise, and the reading of neutral material, respectively. We gave participants in the Baduanjin imagery group a brief introduction to the content of the imagery training. We asked them to wear an eyeshade to reduce light stimulation and enhance visual imagery. Relaxation imagery was given to participants in the Baduanjin imagery group before the Baduanjin imagery to reduce stress and anxiety, thus increasing the effectiveness of the imagery intervention (50). The audio used for the intervention is the style of the State General Administration of Sport of China. The tests were completed again after the intervention. All participants wore fNIRS throughout the experiment to monitor the hemodynamic changes of the PFC.

Before the formal intervention, “Preparation Instructions” was used to tell participants to be ready to enter an intervention. The instructions for the Baduanjin imagery group were: “For the next 15 min, please sit comfortably with an eyeshade, close your eyes and fully participate, do not talk or fall asleep, and do not shake or move your body. Follow the audio for relaxation imagery and Baduanjin imagery. The process is uninterrupted, signal me if you're ready, and we'll start.” The instructions for the Baduanjin exercise group were: “For the next 15 min, please follow the audio for warm-up activities and Baduanjin exercise without interruption throughout. Signal me if you're ready, and we'll start.” For the control group, the instructions were: “For the next 15 min, please have a rest for a while and then read the material I have distributed to you. Signal me if you're ready, and we'll start.” Furthermore, we gave the participants in the Baduanjin imagery group “Baduanjin imagery guidelines” to guide them in forming a visual-motor imagery of the Baduanjin, while the exercise and control groups did not have the guide. According to the “Baduanjin imagery guidelines”, participants should sit in a chair and listen carefully to the Baduanjin movement commands with their eyes closed, follow the commands to imagine as clearly as possible the Baduanjin movements as they had practiced, and “watch” the Baduanjin movement process in their minds like watching a movie.

In addition, the effectiveness of the imagery intervention was assessed by a motor imagery manipulation check (51, 52) after Baduanjin imagery training. The manipulation check consisted of 3 questions by a 5-point Likert scale (5 points represents a more profound feeling) that asked participants to rate their true feelings. The question is as follows: (1) Were you able to visualize the movement during the Baduanjin imagery just now? (2) Was it easy to control the motor imagery during the Baduanjin imagery just now? (3) Was the motor imagery clear and vivid during the Baduanjin imagery just now? In our study, the mean score of the motor imagery manipulation check was 4.29 (SD = 0.41), indicating that the participants developed the Baduanjin imagery with a degree of controllability and clarity above moderate. Therefore, the Baduanjin imagery intervention was effective.

### fNIRS data acquisition

**Figure 3:**
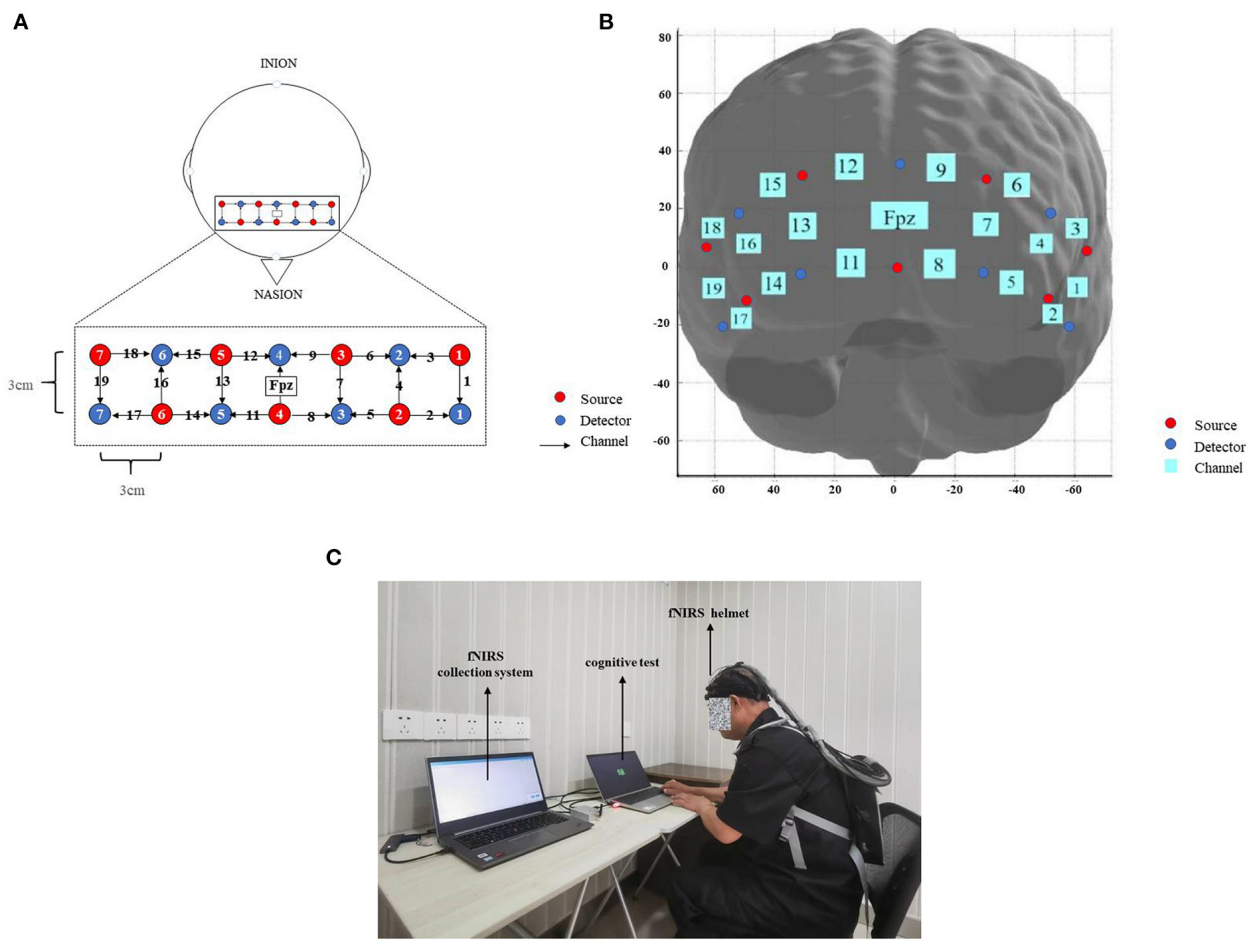
**(A)** A 19-channel functional near-infrared spectroscopy (fNIRS) probe set covering the prefrontal area. **(B)** The center of the probe matrix was placed on Fpz according to the international 10–20 system. **(C)** A figure showing the experimental setup.

The fNIRS data were collected by the NirSmart II system (Danyang Huichuang, China). The fNIRS system uses the 2 × 7 Prefrontal Cortex Layout [Figure 3 (53)] with 19 channels consisting of 7 light source probes and 7 detector probes (probe spacing 3 cm). The sampling rate was 10 Hz. According to the International 10–20 system, the center of the probe was placed on Fpz, covering the prefrontal cortex area of the brain. The standard MNI coordinates are shown in Table 1. The system monitors oxy-hemoglobin (oxy-Hb), deoxy-hemoglobin (deoxy-Hb), and total-hemoglobin (total-Hb) under the subject's prefrontal cortex using two wavelengths of near-infrared light (760 and 850 nm). We calibrated before data collection from each participant, including checking the location of the source-detector separation and ruffling the hair to ensure there was no hair covering the prefrontal lobe.

**Table 1:** The standard MNI coordinates and associated brain areas of the channels.

**Channel**	**MNI coordinate**			**Brodmann area**	**Probability**
	**x**	**y**	**z**		
CH 01	– 53	23	– 7	38—Temporopolar area	0.94
CH 02	– 55	33	18	45—Pars triangularis Broca's area	1
CH 03	– 51	41	– 14	47—Inferior prefrontal gyrus	0.69
CH 04	– 49	49	6	46—Dorsolateral prefrontal cortex	0.69
CH 05	– 40	61	– 6	10—Frontopolar area	0.45
CH 06	– 39	51	27	46—Dorsolateral prefrontal cortex	0.77
CH 07	– 30	65	15	10—Frontopolar area	0.78
CH 08	– 15	61	35	46—Dorsolateral prefrontal cortex	0.50
CH 09	– 15	73	– 1	10—Frontopolar area	0.56
CH 10	– 1	67	18	10—Frontopolar area	1
CH 11	15	73	– 3	10—Frontopolar area	0.45
CH 12	13	63	34	10—Frontopolar area	0.51
CH 13	29	68	14	10—Frontopolar area	0.97
CH 14	40	55	26	46—Dorsolateral prefrontal cortex	0.92
CH 15	41	62	– 9	47—Inferior prefrontal gyrus	0.34
CH 16	50	51	1	46—Dorsolateral prefrontal cortex	0.92
CH 17	52	42	– 16	47—Inferior prefrontal gyrus	0.78
CH 18	58	35	12	45—Pars triangularis Broca's area	1
CH 19	56	19	– 15	38—Temporopolar area 0.94	0.97

### fNIRS data preprocessing

The fNIRS data preprocessing was completed using the NirSpark (Danyang Huichuang, China). The software digitally bandpass filters the original optical density signal (0.01–0.2Hz) to remove common noises including the physiological noises due to heartbeats, respirations, and Mayer waves (54, 55). Using the modified Beer–Lambert's law, the experimentally measured intensity counts at the two wavelengths in all channels were converted to relative-change curves of oxy-Hb, deoxy-Hb, and total-Hb. We used oxy-Hb, which has the highest sensitivity to changes in cerebral blood oxygen (56), to assess the participants' brain activation status. Furthermore, the coordinates of the channels were positioned and aligned with the Brodmann area, where the left DLPFC covered the Channels 4, 6, and 8, and the right IFG covered the Channels 15 and 17.

### Data analysis

Microsoft Office Excel 2019 and IBM SPSS 24.0 were used to analyze the data. The age, BMI, years of Baduanjin exercise, PARS-3 scores, MMSE scores, and MIQ-R scores were assessed by one-way ANOVA. The behavioral results (RTs and ACC) were assessed by a 3 (Group: Baduanjin imagery group, Baduanjin exercise group, and Control group) × 2 (Test phase: Pre-test, Post-test) repeated-measures ANOVA. The oxy-Hb concentrations in the PFC were assessed by a 3 (Group: Baduanjin imagery group, Baduanjin exercise group, and Control group) × 5 (Test phase: Pre-test resting-state, Pre-test task, Intervention, Post-test resting-state, Post-test task) repeated measures ANOVA. The multiple comparisons were performed if there was a main effect. Simple effects analysis was performed if there was an interaction effect between phase and group. Results were tested using the within-subjects effect test if the sphericity assumption was satisfied. Otherwise, the multivariate test results were adopted. The significance level was set as *p* < 0.05.

## Results

### Demographic and scales

Demographic variables, MMSE, PARS-3, and MIQ-R scores are shown in Table 2. There were no significant differences in the scores among the three groups (*ps* > 0.05). The three groups of participants were homogeneities before the experiment.

**Table 2:** Group differences in demographic indicators, MMSE, PARS-3, and MIQ-R (M ± SD).

	**Baduanjin imagery group** **(*n* = 24)**	**Baduanjin exercise group** **(*n* = 25)**	**Control group** **(*n* = 23)**	***F***	***p***	**η^2^**
Age (years)	67.67 ± 4.92	66.72 ± 4.61	66.35 ± 4.89	0.475	0.624	0.014
BMI (kg/m^2^)	25.35 ± 4.12	25.02 ± 4.56	25.77 ± 5.53	0.148	0.862	0.004
Badaunjin exercise experience (years)	7.31 ± 4.68	5.94 ± 3.33	6.17 ± 3.74	0.836	0.438	0.024
Physical activity	29.17 ± 12.94	27.92 ± 13.55	27.39 ± 12.96	0.114	0.893	0.003
MMSE	27.75 ± 1.59	27.68 ± 1.73	28.48 ± 1.31	1.892	0.159	0.052
Kinesthetic Imagery	4.83 ± 1.42	4.65 ± 1.43	4.96 ± 1.20	0.311	0.734	0.009
Visual Imagery	6.29 ± 0.58	6.06 ± 0.75	6.00 ± 0.69	1.226	0.300	0.034

### Behavioral Stroop task

The results of participants' behavioral performance on the cognitive task are shown in Table 3. For the ACC, neither main effects nor interactions were significant under congruent and incongruent tasks (*ps* > 0.05).

**Table 3:** The ACC (%) and RTs (ms) on the Stroop task (M ± SD).

**Index**	**Task type**	**Baduanjin imagery group (*****n*** = **24)**		**Baduanjin exercise group (*****n*** = **25)**		**Control group (*****n*** = **23)**	
		**Pre-test**	**Post-test**	**Pre-test**	**Post-test**	**Pre-test**	**Post-test**
ACC (%)	Congruent task	91.55 ± 5.61	96.87 ± 2.79	92.94 ± 9.44	97.22 ± 3.21	89.01 ± 17.78	92.03 ± 20.80
	Incongruent task	77.78 ± 11.81	87.50 ± 9.30	81.56 ± 15.17	91.28 ± 7.79	78.26 ± 18.38	84.18 ± 13.17
RTs (ms)	Congruent task	845.24 ±110.27	757.45 ± 82.32	835.25 ± 107.55	738.32 ± 93.75	824.53 ± 124.98	740.22 ± 106.62
	Incongruent task	1036.93 ± 117.43	924.29 ± 93.42	1028.88 ± 121.77	894.16 ± 95.68	957.00 ± 124.25	918.65 ± 137.09

For the RTs, the main effect of the test phase was significant under the congruent task, *F* (1, 69) = 112.412, *p* < 0.001, η^2^ = 0.620. The RTs of the post-test (745.31 ± 93.61 ms) was significantly shorter than the pre-test (835.16 ± 112.96 ms). The main effect of the group and the interaction between phase and group were not significant (*ps* > 0.05) under the congruent task. Under the incongruent task, the main effect of the group was not significant (*p* = 0.411). The main effect of the test phase was significant, *F* (1, 69) = 114.076, *p* < 0.001, η^2^ = 0.623. The RTs of the post-test (912.02 ± 109.19 ms) was significantly faster than the pre-test (1008.58 ± 124.66 ms). The interaction between the test phase and group was statistically significant, *F* (2, 69) = 10.533, *p* < 0.001, η^2^ = 0.234. The simple effect analysis further demonstrated that the RTs of participants in the Baduanjin imagery group [*t* (23) = 7.595, *p* < 0.001] and Baduanjin exercise group [*t* (24) = 12.830, *p* < 0.001] were significantly faster in the post-test than in the pre-test, However, there was no significant difference between the pre-test and the post-test (*p* = 0.071) for the Control group. In addition, there were no between-group differences in both the pre-test and post-test (*ps* > 0.05). These results indicate that the Baduanjin imagery and Baduanjin exercise significantly shortened participants' reaction time to complete an inhibitory control task and increased practitioners' cognitive response speed.

### fNIRS results

**Figure 4:**
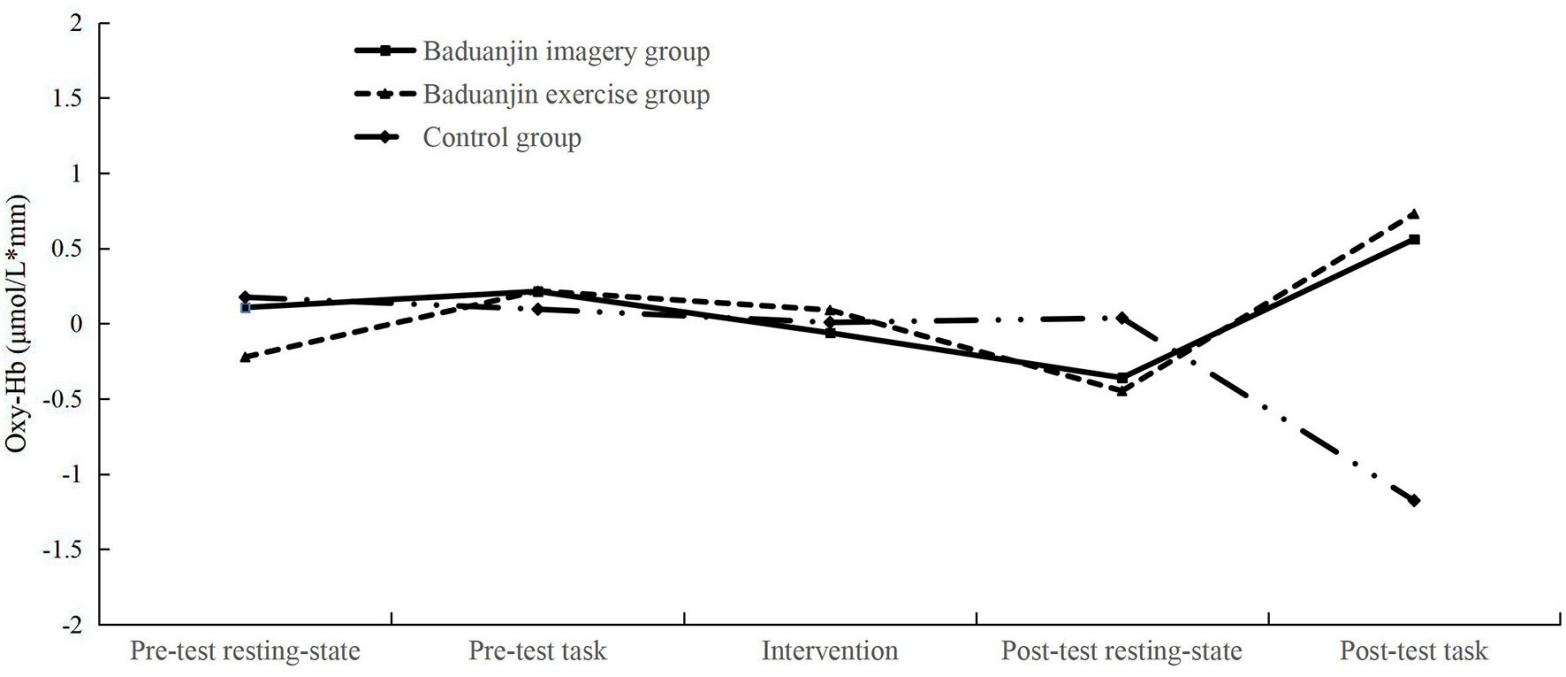
In the incongruent task, the mean oxy-Hb concentration variations of the left DLPFC for the three groups of participants (μmol/L × mm). After the intervention, the oxy-Hb variations were significantly higher in participants of the Baduanjin imagery group and Baduanjin exercise group than in the Control group.

We analyzed the oxy-Hb data in the PFC measured by fNIRS. For the left DLPFC, the result demonstrated that the main effect of the test phase, the main effect of the group, and the interaction between the test phase and group were not significant in the congruent task (*ps* > 0.05). In the incongruent task, the main effects of the test phase and group were not significant (*ps* > 0.05). The interaction between the test phase and group was significant, *F* (2, 69) = 2.554, *p* = 0.013, η^2^ = 0.132. The results of the simple effect test showed that there were significant differences in the post-test task among the three groups, *F* (2, 69) = 6.254, *p* = 0.003, η^2^ = 0.153. The oxy-Hb variations in the left DLPFC in participants of the Baduanjin imagery group (*p* = 0.005) and Baduanjin exercise group (*p* = 0.002) were significantly higher than that in the Control group. In the pre-test task, there was no significant difference between participants of the three groups (*p* = 0.965). The oxy-Hb concentrations of participants in three groups at all five phases were not significant (*ps* > 0.05). These results indicate that the Baduanjin imagery and the Baduanjin exercise significantly increased the oxy-Hb concentration in the participants' left DLPFC in the post-test cognitive state (Figure 4). See Supplementary Table 2 for specific oxy-Hb signals of all groups.

**Figure 5:**
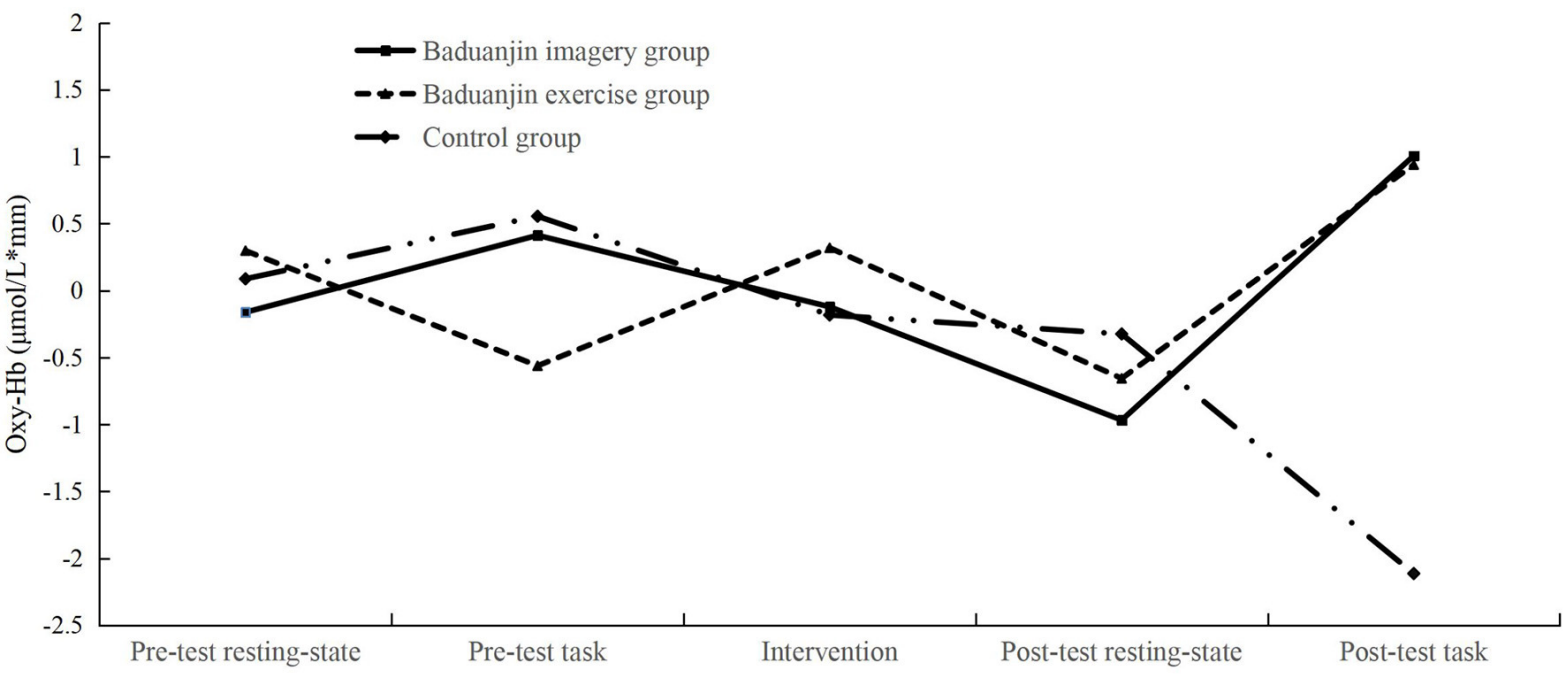
In the incongruent task, the mean oxy-Hb concentration variations of the right IFG for the three groups of participants (μmol/L × mm). After the intervention, the oxy-Hb variations were significantly higher in participants of the Baduanjin imagery group than in the Control group.

For the right IFG, the main effect of the test phase, the main effect of the group, and the interaction between the test phase and the group were not significant in the congruent task (*ps* > 0.05). In the incongruent task, the main effect of the test phase was not significant (*ps* > 0.05); the main effect of the group was significant, *F* (2, 69) = 3.356, *p* = 0.041, η^2^ = 0.089. The multiple comparison results showed that the oxy-Hb variation of the Baduanjin imagery group was significantly higher than that in the Control group (*p* = 0.016). The interaction between the test phase and group was significant, *F* (2, 69) = 2.060, *p* = 0.044, η^2^ = 0.110. The simple effect test showed significant differences in the post-test task among the three groups, *F* (2, 69) = 5.933, *p* = 0.004, η^2^ = 0.147. The oxy-Hb variation in the right IFG in participants of the Baduanjin imagery group was significantly higher than that in the Control group (*p* = 0.001), while not in the Baduanjin exercise group (*p* = 0.069). In the pre-test task, there was no significant difference between participants of the imagery and control groups (*p* = 0.665). The oxy-Hb concentrations of participants in three groups at all five phases were not significant (*ps* > 0.05). These results indicate that the Baduanjin imagery significantly increased the oxy-Hb concentration in the participants' right IFG in the post-test cognitive state (Figure 5). See Supplementary Table 3 for specific oxy-Hb signals of all groups. Other brain areas' results are detailed in the Supplemental material.

## Discussion

Our study aimed to examine the effects of traditional Chinese health exercises Baduanjin imagery and exercise on cognitive brain function in the elderly. The research suggests that the Baduanjin imagery and the Baduanjin exercise can improve the elderly' performance in the Stroop task and promote brain activation in the left DLPFC during the inhibitory control task. In addition, Baduanjin imagery was also shown to activate the right IFG.

The results of the Stroop task suggested that there was no significant difference in the RTs of the participants in the pre-test. However, as predicted, the RTs of the Baduanjin imagery and exercise groups in the incongruent task were significantly shorter after the intervention, indicating that the cognitive reaction speed of the elderly in both groups was significantly improved, which supports the findings of the research related to the Baduanjin exercise. For example, Chen et al. (17) conducted that the Baduanjin exercise intervention significantly reduced RTs to complete Stroop tasks in college students and contributed to improved executive function.

The present study also supports the findings of previous research related to motor imagery. A cross-sectional study conducted by Pelletier et al. (42) found a significant correlation between Left Right Judgement Task (LRJT) performance and Stroop task reaction time. The LRJT task involves motor imagery that reflects individual motor imagery ability, and the Stroop task reflects individual inhibitory control ability. Thus, this study suggests that motor imagery level is correlated with the reaction time of individuals completing the Stroop task. Our research results further indicate that motor imagery interventions can improve subjects' reaction speed in completing the inhibitory control task. In addition, the present study supports the finding of Blumen et al. (41) that motor imagery can shorten participants' reaction time to the cognitive task.

However, the results of the Stroop task showed that there was no significant difference in participants' ACC before and after the intervention. Maybe the task was too simple, or participants were too motivated to do the task correctly, resulting in no significant improvement in ACC after the intervention. A study of the effect of exercise on cognitive control ability in college students conducted by Li et al. (9) also found that the movement did not significantly improve participants' ACC of the Stroop task but instead had an increased effect on reaction time indicators. This suggests that improvements in inhibitory control functions may be reflected mainly in RTs rather than changes in ACC (9, 10).

The fNIRS results showed that the Baduanjin exercise significantly increased the left DLPFC oxy-Hb in older adults when completing inhibitory control tasks. This result supports the previous related studies. A study of the effects of Baduanjin exercise on brain function in the elderly conducted by Jiang et al. (57) found that older adults who practiced Baduanjin for a long time had greater prefrontal activation and higher blood oxygen concentration. Moreover, Zheng et al. (58) found by fMRI that the Baduanjin exercise improved blood flow changes in brain regions associated with cognitively impaired elderly. Their study showed that the Baduanjin exercise improved blood flow levels in brain regions associated with cognitive function in the elderly (59). Similarly, the present study found an increase in oxy-Hb concentrations in the left DLPFC of older adults after the Baduanjin exercise, suggesting that the Baduanjin exercise induces changes in brain function in older adults.

However, the right IFG was possibly less sensitive compare to the left DLPFC. The results of the fNIRS showed that the right IFG was not activated by the Baduanjin exercise, which is different from the results of previous studies (30–32), which may be due to the different exercise types and intensities of exercises used. The sports types in previous research were primarily strategic sports (31, 32), and the activation of right IFG was found by controlling the exercise intensity to moderate intensity. Studies have shown that brain activation in the elderly was affected by exercise intensity, with higher exercise intensities (higher VO_2max_) more likely to contribute to increased cerebral oxygenation in the elderly (60, 61). In contrast, the Baduanjin exercise used in the present study was a closed-loop exercise with low intensity, which may lead to a non-significant activation effect after the intervention.

More importantly, the fNIRS results showed that the Baduanjin imagery successfully activated the left DLPFC neurons in the elderly when completing cognitive tasks, increasing oxy-Hb in this brain region. Since elevated oxy-Hb concentrations imply enhanced cortical activation, the Baduanjin imagery training enhances activation of the left DLPFC during the inhibitory control task. Motor imagery has remarkably similar neural substrates to actual movement, activating the frontal and parietal lobes (29, 62). Zhang (63) and Kotegawa (64) found that motor imagery significantly increased participants' PFC blood oxygen levels and had some activating effect on DLPFC. The present study demonstrated that the Baduanjin imagery could induce changes in blood concentration in the left DLPFC, which was beneficial for improving brain function during cognitive tasks in older adults, further supporting previous motor imagery-related studies (17, 63). In addition, the results demonstrated that the Baduanjin imagery activated the right IFG, which supports the previous studies (33–35). The IFG is part of the mirror neuron system, which may play an essential role in motor imagery (35, 65, 66). Studies have shown that motor imagery is associated with the activation of the mirror neuron system (67) and can change the function of the cortical areas (68). This may explain why the right IFG blood concentration increased in the elderly during the post-test task after the Baduanjin imagery.

The DLPFC and the IFG are the functional center of higher cognitive activities concerning attention (69), working memory (70), and cognitive control (24, 26). The activation of those region, a critical region of the cognitive brain network, can serve as the neural basis for improving inhibition and control ability. We suggest that the underlying mechanism of Baduanjin imagery and exercise might be the activation of the left DLPFC and the right IFG.

We noticed that the control group showed negative oxygenation in the post-test tasks. It is possibly due to the suspension of the neurovascular coupling (71). The cognitive fatigue caused by the continuous completion of cognitive tasks could trigger a pause in neurovascular coupling in the elderly (71–73), which may lead to a decrease in the neuronal firing rate and a reduced blood flow, exhibiting a decrease in oxy-Hb during the post-test task. On the contrary, the intervention of the Baduanjin imagery and exercise activated neurons in the elderly and increased the oxygenation of the brain area in the post-test task, indicating the positive effects of the Baduanjin imagery and exercise.

Our research showed that Baduanjin imagery training could improve the cognitive function of the elderly similar to the actual movement, highlighting the positive effect of motor imagery. In the past, motor imagery was mainly used to improve skill learning effect and physical motor function, and the target groups were primarily teenagers and healthy adults. However, as research has intensified, motor imagery has gradually become a novel non-physical means of improving physical and mental health in the elderly (74). With the widespread Baduanjin movement, the Baduanjin imagery practice has more advantages in an application. For example, (1) Baduanjin imagery has a unique cultural component compared with the imagery training of traditional sports, making the exercise less dull and more attractive; (2) The Baduanjin movement has only eight simple actions that are highly popularized. Therefore, the Baduanjin imagery has the advantages of being simple and easy to learn, which is conducive to popularization and application; (3) Baduanjin imagery has a positive effect on the recovery of neurological diseases and can improve the cognitive rehabilitation effect of the elderly patients, such as cerebral stroke (75, 76) and Parkinson's (20) patients; (4) Under the background of the epidemic (COVID-19), motor imagery training is not limited by external factors such as venue, equipment, weather, so it can be used as a simple way of home-based exercise. Baduanjin imagery training, such as watching videos and imaging actions, can alleviate the brain aging decline and improve the cognitive function of people living at home.

There are some shortcomings in this study that need to be further improved in the future. First, this study only monitored oxy-Hb changes in the PFC. We can continue to explore the effects of the Baduanjin imagery on blood oxygen concentration in other brain regions. Secondly, the study used visual-motor imagery for the intervention, which required participants to form clear pictures of the movement process. Still, the imagery can be divided into first-person, third-person, and kinesthetic, depending on the perspective and media. Subsequently, whether different types of motor imagery have the same brain activation effect still needs to be investigated. Thirdly, the study lacked the short-separation channel due to the limitation of the instrument utilized, which may cause the interference of superficial cortex blood flow. In order to ensure the scientific effectiveness of the research, a more refined fNIRS should be used for research in the future. Finally, this study used a traditional Chinese health exercise, Baduanjin, as an intervention program and found similar brain activation effects between motor imagery and exercise in the elderly. Future studies can further examine the impacts of motor imagery training in other traditional Chinese health exercises on cognitive function in the elderly.

## Conclusion

Baduanjin imagery and exercise can shorten the reaction time of inhibitory control tasks; Baduanjin imagery and exercise activated the left dorsolateral prefrontal cortex; Baduanjin imagery activated the right inferior frontal gyrus, while Baduanjin exercise could not. The Baduanjin imagery as a mental exercise modality could be a convenient and safe intervention to enhance cognitive function in the elderly.

## Data availability statement

The datasets presented in this study can be found in online repositories. The names of the repository/repositories and accession number(s) can be found in the article/Supplementary material.

## Ethics statement

The studies involving human participants were reviewed and approved by the Ethics Committee of the School of Public Health of Shandong University. The patients/participants provided their written informed consent to participate in this study. Written informed consent was obtained from the individual(s) for the publication of any potentially identifiable images or data included in this article.

## Author contributions

LY and YH provided the ideas. LY participated in writing the introduction, methods, results, discussion, the experiments' conducting, and the data processing. JW participated in the revisions of the article. GS completed the editing and review of the article. All authors contributed to the article and approved the submitted version.

## Funding

This study was funded by the Social Science Fund of Shandong Province (No. 22CTYJ07), the Young Scholars Program of Shandong University, the Teaching Research Project of Shandong University (No. XYJG2020025), and the Graduate Research Fund of Shandong University College of Physical Education (No. 2021YJKTYB29).

## Conflict of interest

The authors declare that the research was conducted in the absence of any commercial or financial relationships that could be construed as a potential conflict of interest.

## Publisher's note

All claims expressed in this article are solely those of the authors and do not necessarily represent those of their affiliated organizations, or those of the publisher, the editors and the reviewers. Any product that may be evaluated in this article, or claim that may be made by its manufacturer, is not guaranteed or endorsed by the publisher.
