# Supplementary material for: Effects of Baduanjin imagery and exercise on cognitive function in the elderly: A functional near-infrared spectroscopy study
Source: Front Public Health. 2022 Sep 29;10:968642. doi: 10.3389/fpubh.2022.968642 (PMC9557749; doi:10.3389/fpubh.2022.968642)
Supplement: Supplementary file 1 [file Data_Sheet_1.docx]

***Supplementary Material***

The fNIRS data preprocessing was completed using the NirSpark (Danyang Huichuang, China). The detailed process is the same as the body of the text. Furthermore, the coordinates of the channels were positioned and aligned with the Brodmann area, and the corresponding channels of the other brain areas in the prefrontal cortex are as follows (Supplementary Table 1).

In the data analysis, the oxy-Hb concentrations in the other brain areas were assessed by a 3 (Group: Baduanjin imagery group, Baduanjin exercise group, and Control group) × 5 (Test phase: Pre-test resting-state, Pre-test task, Intervention, Post-test resting-state, Post-test task) repeated measures ANOVA. The multiple comparisons were performed if there was a main effect. Simple effects analysis was performed if there was an interaction effect between phase and group. Results were tested using the within-subjects effect test if the sphericity assumption was satisfied. Otherwise, the multivariate test results were adopted. The significance level was set as *p* < 0.05.

**1 Supplementary Data**

We analyzed the oxy-Hb data in the other brain areas measured by fNIRS. The result demonstrated that the main effect of the test phase, the main effect of the group, and the interaction between the test phase and the group were not significant in the congruent task (*ps* > 0.05).

In the incongruent task, for the right temporopolar area, the main effect of the test phase was not significant (*ps* > 0.05); the main effect of the group was significant, *F* (2, 69) = 3.431, *p* = 0.038, *η*^2^ = 0.090. The multiple comparison results showed that the oxy-Hb variation of the Baduanjin imagery group was significantly higher than that in the Control group (*p* = 0.014). The interaction between the test phase and group was significant, *F* (2, 69) = 2.229, *p* = 0.029, *η*^2^ = 0.117. The results of the simple effect test showed that there were significant differences in the intervention, *F* (2, 69) = 3.175, *p* = 0.048, *η*^2^ = 0.084. The oxy-Hb variation in the right temporopolar area in the Baduanjin exercise group was significantly higher than that in the Control group (*p* = 0.015). There were also significant differences in the post-test task, *F* (2, 69) = 8.356, *p* = 0.001, *η*^2^ = 0.195. The oxy-Hb variations in the Baduanjin imagery group (*p* < 0.001) and Baduanjin exercise group (*p* = 0.002) were significantly higher than that in the Control group. In the pre-test task, there was no significant difference between participants of the three groups (*p* = 0.592) (Supplementary Table 4).

For other brain areas, there were no significant interactions in the incongruent task (*ps* > 0.05) (Supplementary Table 5-11).

**2 Supplementary Tables**

**Supplementary Table 1**. Correspondence between brain areas and channels.

|  | Channel |
| --- | --- |
| Right dorsolateral prefrontal cortex | CH14, CH16 |
| Left Inferior prefrontal gyrus | CH3 |
| Left frontopolar area | CH5, CH7, CH9 |
| Right frontopolar area | CH11, CH12, CH13 |
| Left pars triangularis Broca's area | CH2 |
| Right pars triangularis Broca's area | CH18 |
| Left temporopolar area | CH1 |
| Right temporopolar area | CH19 |

**Supplementary Table 2**. In the incongruent task, the oxy-Hb of the left dorsolateral prefrontal cortex for the three groups of participants (*M* ± *SD*).

| Group | Pre-test  resting-state | Pre-test task | Intervention | Post-test resting-state | Post-test task |
| --- | --- | --- | --- | --- | --- |
| Baduanjin imagery group | 0.107 ± 0.7100 | 0.214 ± 1.7075 | -0.062 ± 0.1513 | -0.360 ± 1.1500 | 0.561 ± 1.5606 |
| Baduanjin exercise group | -0.222 ± 0.6262 | 0.217 ± 1.6531 | 0.090 ± 0.7335 | -0.447 ± 2.8771 | 0.730 ± 2.5160 |
| Control group | 0.175 ± 0.8665 | 0.095 ± 1.9832 | 0.008 ± 0.4453 | 0.037 ± 1.0850 | -1.176 ± 1.9183 |

**Supplementary Table 3**. In the incongruent task, the oxy-Hb of the right inferior prefrontal gyrus for the three groups of participants (*M* ± *SD*).

| Group | Pre-test  resting-state | Pre-test task | Intervention | Post-test resting-state | Post-test task |
| --- | --- | --- | --- | --- | --- |
| Baduanjin imagery group | -0.065 ± 0.8309 | 0.906 ± 3.6126 | -0.058 ± 0.2496 | 0.507 ± 3.1023 | 1.413 ± 2.3781 |
| Baduanjin exercise group | 0.266 ± 1.4245 | -0.223 ± 3.5667 | 0.016 ± 0.7738 | -0.406 ± 2.2559 | -0.098 ± 3.6744 |
| Control group | 0.231 ± 0.6488 | 0.472 ± 3.0240 | 0.140 ± 0.4854 | -0.418 ± 1.3904 | -1.804 ± 3.3915 |

**Supplementary Table 4**. In the incongruent task, the oxy-Hb of the right temporopolar area for the three groups of participants (*M* ± *SD*).

| Group | Pre-test  resting-state | Pre-test task | Intervention | Post-test  resting-state | Post-test task |
| --- | --- | --- | --- | --- | --- |
| Baduanjin imagery group | 0.656 ± 2.9978 | 0.030 ± 6.6997 | 0.028 ± 0.7031 | -0.882 ± 2.8870 | 0.770 ± 5.5070 |
| Baduanjin exercise group | -0.127 ± 2.9100 | -2.126 ± 5.6689 | 0.370 ± 1.8282 | -0.184 ± 10.8327 | -0.139 ± 4.3271 |
| Control group | -0.141 ± 1.6822 | -1.67 ± 10.1584 | -0.566 ± 1.0513 | -1.993 ± 3.3545 | -6.023 ± 8.2122 |

**Supplementary Table 5**. In the incongruent task, the oxy-Hb of the right dorsolateral prefrontal for the three groups of participants (*M* ± *SD*).

| Group | Pre-test  resting-state | Pre-test task | Intervention | Post-test resting-state | Post-test task |
| --- | --- | --- | --- | --- | --- |
| Baduanjin imagery group | -0.032 ± 0.9813 | 0.051 ± 2.6114 | -0.018 ± 0.2567 | -0.011 ± 2.3454 | 0.488 ± 2.2200 |
| Baduanjin exercise group | 0.090 ± 1.0350 | -0.320 ± 4.4184 | -0.143 ± 0.5669 | -0.725 ± 2.4708 | -0.735 ± 4.0191 |
| Control group | -0.159 ± 1.1143 | 0.120 ± 3.7640 | 0.085 ± 0.3125 | -0.539 ± 1.5733 | -1.203 ± 3.0099 |

**Supplementary Table 6**. In the incongruent task, the oxy-Hb of the left inferior prefrontal gyrus for the three groups of participants (*M* ± *SD*).

| Group | Pre-test  resting-state | Pre-test task | Intervention | Post-test resting-state | Post-test task |
| --- | --- | --- | --- | --- | --- |
| Baduanjin imagery group | -0.162 ± 1.4207 | 0.412 ± 5.3395 | -0.121 ± 0.3774 | -0.968 ± 2.8108 | 1.006 ± 2.6630 |
| Baduanjin exercise group | 0.298 ± 3.1460 | -0.562 ± 5.2635 | 0.317 ± 1.1447 | -0.656 ± 2.7433 | 0.940 ± 3.7276 |
| Control group | 0.087 ± 1.8012 | 0.555 ± 5.5683 | -0.183 ± 0.4936 | -0.324 ± 1.3539 | -2.114 ± 3.8408 |

**Supplementary Table 7**. In the incongruent task, the oxy-Hb of the left frontopolar area for the three groups of participants (*M* ± *SD*).

| Group | Pre-test  resting-state | Pre-test task | Intervention | Post-test resting-state | Post-test task |
| --- | --- | --- | --- | --- | --- |
| Baduanjin imagery group | 0.204 ± 0.5849 | 0.488 ± 2.0543 | -0.065 ± 0.1676 | -0.217 ± 1.5744 | 0.279 ± 1.9114 |
| Baduanjin exercise group | 0.051 ± 0.8950 | 0.055 ± 3.3158 | -0.253 ± 1.1667 | -0.019 ± 1.6596 | -0.040 ± 2.3021 |
| Control group | 0.326 ± 0.8933 | -0.145 ± 2.9565 | 0.183 ± 0.3733 | 0.130 ± 0.9849 | -1.234 ± 3.1837 |

**Supplementary Table 8**. In the incongruent task, the oxy-Hb of the right frontopolar area for the three groups of participants (*M* ± *SD*).

| Group | Pre-test  resting-state | Pre-test task | Intervention | Post-test resting-state | Post-test task |
| --- | --- | --- | --- | --- | --- |
| Baduanjin imagery group | 0.156 ± 0.7245 | 0.409 ± 2.3635 | -0.075 ± 0.2229 | -0.390 ± 1.7132 | 0.103 ± 1.9320 |
| Baduanjin exercise group | 0.009 ± 0.6658 | -0.313 ± 2.8224 | -0.322 ± 0.7469 | -0.094 ± 2.0610 | -0.523 ± 2.8571 |
| Control group | 0.329 ± 0.9030 | -0.025 ± 5.4082 | -0.061 ± 0.7186 | 0.280 ± 1.1594 | -1.079 ± 2.8162 |

**Supplementary Table 9**. In the incongruent task, the oxy-Hb of the left pars triangularis Broca's area for the three groups of participants (*M* ± *SD*).

| Group | Pre-test  resting-state | Pre-test task | Intervention | Post-test resting-state | Post-test task |
| --- | --- | --- | --- | --- | --- |
| Baduanjin imagery group | -0.180 ± 1.2395 | 0.360 ± 4.2776 | 0.048 ± 0.2414 | 0.197 ± 2.3875 | 0.308 ± 3.4448 |
| Baduanjin exercise group | -0.134 ± 1.2739 | 1.386 ± 5.8304 | 0.256 ± 1.3267 | 0.372 ± 3.2662 | 0.450 ± 3.4628 |
| Control group | 0.594 ± 1.2099 | -0.617 ± 3.5800 | 0.364 ± 0.7841 | 0.646 ± 2.9461 | -0.095 ± 3.3751 |

**Supplementary Table 10**. In the incongruent task, the oxy-Hb of the right pars triangularis Broca's area for the three groups of participants (*M* ± *SD*).

| Group | Pre-test  resting-state | Pre-test task | Intervention | Post-test resting-state | Post-test task |
| --- | --- | --- | --- | --- | --- |
| Baduanjin imagery group | 0.197 ± 1.2463 | 0.023 ± 3.2587 | -0.015 ± 0.4040 | 0.133 ± 2.0421 | 0.390 ± 4.3572 |
| Baduanjin exercise group | -0.215 ± 3.0695 | -0.316 ± 4.2227 | 0.222 ± 0.9738 | -0.360 ± 4.7206 | -1.502 ± 4.5899 |
| Control group | 0.111 ± 1.4633 | -1.002 ± 5.1089 | 0.215 ± 0.3666 | 0.274 ± 1.4362 | -1.669 ± 2.7581 |

**Supplementary Table 11**. In the incongruent task, the oxy-Hb of the left temporopolar area for the three groups of participants (*M* ± *SD*)

| Group | Pre-test  resting-state | Pre-test task | Intervention | Post-test resting-state | Post-test task |
| --- | --- | --- | --- | --- | --- |
| Baduanjin imagery group | -0.136 ± 1.7863 | -0.137 ± 5.4275 | 0.006 ± 0.4201 | -1.175 ± 4.2750 | -0.485 ± 5.0241 |
| Baduanjin exercise group | -0.109 ± 2.2401 | 1.666 ± 12.3830 | 0.220 ± 1.7559 | -1.433 ± 4.4503 | 0.371 ± 4.6415 |
| Control group | 0.241 ± 1.6440 | -1.024 ± 5.9871 | -0.005 ± 0.4944 | -0.159 ± 2.7538 | -0.041 ± 3.5932 |
